# Supplementary material for: A catalog of CasX genome editing sites in common model organisms
Source: BMC Genomics. 2019 Jun 27;20:528. doi: 10.1186/s12864-019-5924-6 (PMC6598274; doi:10.1186/s12864-019-5924-6)
Supplement: Supplementary file 1 — Table S1. GC PAM sites depleted in reference genomes (DOCX 15 kb) [file 12864_2019_5924_MOESM1_ESM.docx]

| Supp. Table 1 - GC PAM sites depleted in reference genomes   \| **Species** \| **Diff** \| **Genome GC** \| **estimate** \| **Pvalue** \| **FDR pvalue** \| \| --- \| --- \| --- \| --- \| --- \| --- \| \| *S. cerevisiae* \| LessThanExpected \| 0.3815 \| 0.3518 \| 1.36e-303 \| 4.07e-303 \| \| *C. elegans* \| GreaterOrEqualToExpected \| 0.3544 \| 0.3646 \| 1.00e+00 \| 1.00e+00 \| \| *D. melanogaster* \| GreaterOrEqualToExpected \| 0.4201 \| 0.4472 \| 1.00e+00 \| 1.00e+00 \| \| *D. rerio* \| LessThanExpected \| 0.3665 \| 0.2636 \| 0.00e+00 \| 0.00e+00 \| \| *M. musculus* \| LessThanExpected \| 0.4167 \| 0.2998 \| 0.00e+00 \| 0.00e+00 \| \| *R. norvegicus* \| LessThanExpected \| 0.4193 \| 0.3067 \| 0.00e+00 \| 0.00e+00 \| \| *H. sapiens* \| LessThanExpected \| 0.4086 \| 0.2875 \| 0.00e+00 \| 0.00e+00 \| |
| --- | --- | --- | --- | --- | --- | --- | --- | --- | --- | --- | --- | --- | --- | --- | --- | --- | --- | --- | --- | --- | --- | --- | --- | --- | --- | --- | --- | --- | --- | --- | --- | --- | --- | --- | --- | --- | --- | --- | --- | --- | --- | --- | --- | --- | --- | --- | --- | --- |

Genome GC was calculated based on the genome FASTA file. The estimate is the binomial test estimate of actual C/G usage in the PAM sites, with the associated p-value and the Benjamini Hochberg FDR p-value.
